# Financial Autonomy Versus Financial Safety: A Necessary Tradeoff?

**DOI:** 10.1093/geroni/igaf122.656

**Published:** 2025-12-31

**Authors:** Marguerite DeLiema

**Affiliations:** University of Minnesota School of Social Work, Minneapolis, Minnesota, United States

## Abstract

Persistent and chronic fraud victims may not recognize the harmful consequences of responding to scams, placing them at risk of continued victimization. An adult protection law in Minnesota authorizes financial institutions to place 3-week holds on transactions if an older adult is believed to be experiencing financial abuse or fraud. Many states have recently passed similar laws, but none have systematically collected data on temporary account holds and how they impact older adults’ financial safety and overall well-being. In this study, researchers partnered with the Minnesota Department of Commerce and Adult Protective Services to analyze data on elder financial exploitation cases that were referred for temporary account holds between 2022 and 2024. Researchers then interviewed 12 older adults who experienced temporary account holds, or their designated proxies, to understand the material and psychological effects of the intervention. In 2024 there were 638 investigations opened following referrals for temporary account holds (mean = 53 cases/month); double the number from 2022. Temporary account holds were implemented in 19% of the investigations, resulting in the protection of $8 million dollars. Interview participants described how the perpetrators were not able to take their money during the hold period, but expressed anger and mistrust toward their financial institutions for placing the holds. Results indicate that temporary holds can protect chronic victims against significant financial losses, but when applied too broadly to funds in an account or placed without sufficient communication, account holds can cause significant psychological distress and some material harm.

